# Colorectal mucinous adenocarcinoma indicates a meaningful subtype: A whole genome sequencing study

**DOI:** 10.1002/ctm2.1246

**Published:** 2023-04-26

**Authors:** Yunhua Xu, Xiguang Chen, Yuqiao Chen, Xiaofeng Wu, Qing Fang, Xiangwen Tan, Shuxiang Li, Qiulin Huang, Xuyu Zu, Kai Fu, Shuai Xiao

**Affiliations:** ^1^ The First Affiliated Hospital Cancer Research Institute Hengyang Medical School University of South China Hengyang P. R. China; ^2^ The First Affiliated Hospital Institute of Clinical Medicine Hengyang Medical School University of South China Hengyang P. R. China; ^3^ Institute of Molecular Precision Medicine and Hunan Key Laboratory of Molecular Precision Medicine Xiangya Hospital Central South University Changsha P. R. China; ^4^ The First Affiliated Hospital Department of Gastrointestinal Surgery Hengyang Medical School University of South China Hengyang P. R. China

Dear Editor,

Colorectal cancer (CRC) is a common lethal gastrointestinal tumour. Mucinous adenocarcinoma (MAC) is a special histological subtype of CRC, which characterized by abundant extracellular mucin.^1^ MAC has distinct characteristics compared with the commonest subtype, non‐specific adenocarcinoma (AC), including clinicopathologic factors, molecular features, therapy response, and prognosis.^2^, ^3^, ^4^, ^5^ However, since the molecular mechanisms differences between the MAC and AC are still unclear, all of the current treatment guidelines rarely notice their distinction.^6^, ^7^

To illustrate these issues, we firstly performed RNA‐sequencing on 40 samples comprising 15 MACs, 15 ACs, and 10 normal colorectal tissues, which were collected from our central (hereinafter referred as USC set). The baseline feature of CRC cases is appeared in Table S1. Through the clinicopathological features analysis, we found MAC had a more proximal colon location (*p* < .05) and larger tumour size (*p* < .05). In addition, MAC showed a higher pT stage and more frequent dMMR tendency (Table 1). Then, the TCGA‐COAD transcriptome and clinical data (referred as TCGA set) were also downloaded and analyzed. Through analyzing TCGA clinical data, we got a similar result: no significant difference in other items except dMMR (Table S2).

**TABLE 1:** The baseline characteristics of MAC and AC patients.

	AC (cases)	MAC (cases)	*p* Value
**Sex**			
Female	12	8	
Male	3	7	.245
**Age (years)**	62.00 ± 8.08	61.00 ± 13.62	.809
**Location**			
Proximal	4	11	
Distal	8	2	
Rectal	3	2	.029
**Size (cm)**	5.10 ± 1.64	7.07 ± 2.27	.011
**pT stage**			
0‐2	4	1	
3‐4	11	14	.330
**pN stage**			
N0	13	12	
N+	2	3	1.000
**MMR status**			
pMMR	12	10	
dMMR	1	3	
NA	2	2	.541
**MVI/PNI status**			
Absent	14	13	
Present	1	2	1.000

Abbreviations: AC, adenocarcinoma; dMMR, deficient of MMR; EMT, epithelial‐mesenchymal transition; GEO, Gene Expression Omnibus; GSEA, Gene Set Enrichment Analysis; MAC, mucinous adenocarcinoma; MMR, mismatch repair gene; MSI, microsatellite instability; MSS, microsatellite stable; MVI/PNI, microvascular invasion/perineural invasion; MYC, MYC Proto‐Oncogene; NA, not available; pMMR, proficient of MMR; SM, supplementary material; TCGA, The Cancer Genome Atlas; TGF, transforming growth factor; TTN, Titin; USC, University Of South China.

To elucidate the genome characteristic between MAC and AC, we analyzed the TCGA‐COAD mutation data. Results showed that the mutation atlas of them was notably different. In the AC group, APC (74.6%) had the highest mutation frequency followed by TP53 (59.0%), TTN (49.4%) (Figure 1A). While in the MAC group, TTN (72.4%), APC (65.5%), and KRAS (46.6%) are the most mutated genes (Figure 1B). Additionally, we observed a high mutation frequency of BRAF, whereas TP53 was rare in MAC (Figure 1A, B). Additionally, comparing the common 10 mutant genes, we found only the mutation rate of TTN, RYR2, and OBSCN were distinctly different (Figure 1C). In addition, we observed that the integral genome alteration frequency of MAC is higher than AC (Figure 1D). Finally, we compared the fraction genome alteration of the above three genes. Although the mutation rate of these genes was higher in MAC, the fraction genome alteration was lower in MAC, and each gene had a unique mutational feature (Figure 1E).

**FIGURE 1:**
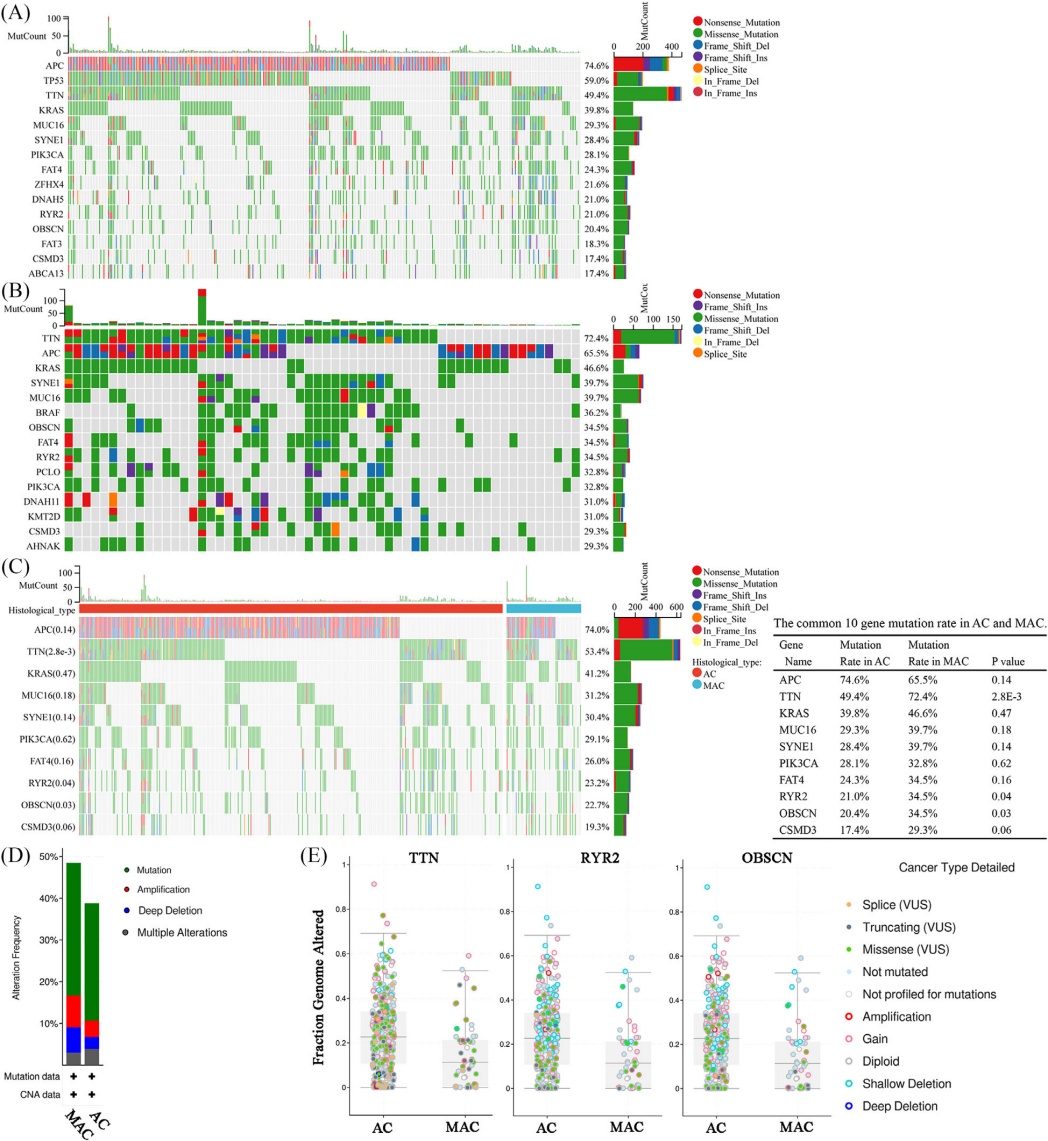
The mutation signature of adenocarcinoma (AC) and mucinous adenocarcinoma (MAC). (A and B) The genomic landscape shows the top 15 mutated genes among AC, MAC. (C) The comparison of common 10 genes mutations in AC and MAC. The values in brackets indicate the statistical value in the mutation frequency of each gene between the two groups. Each column denotes an individual tumour, and each row represents the individual genes. The mutation rate of each gene in all samples was shown in right. (D) The comparison of the integral genome alteration frequency among AC, MAC. (E) The fraction genome altered of TTN, RYR2, and OBSCN. Colours indicate the type of genetic alterations as indicated in the legend.

To study the transcriptomics features of MAC and AC, we performed differentially expressed genes (DEGs) analysis in TCGA and USC sets, respectively. In the TCGA set, we found thousands of DEGs between tumour and normal tissue (Figure 2A,B), and hundreds of DEGs between MAC and AC (Figure 2C). In the USC set, we got similar results (Figure 2D–F). The distribution of DEGs was shown as the ternary plot. The variation was apparent among transcriptional properties of different subtypes (Figure 2G,H). Finally, differences and similarities of DEGs for the TCGA and USC datasets are summarized in Figure 2I. Furthermore, we synthesize intersection DEGs of MAC versus AC in USC and TCGA set and performed functional enrichment analysis to uncover the potential gene functions. Through GO, KEGG, cancer hallmarks, and Reactome analysis, we found the gene functions of these DEGs were correlated with symporter activity, and metabolism‐related molecules and pathways (SM1, Figure S1).

**FIGURE 2:**
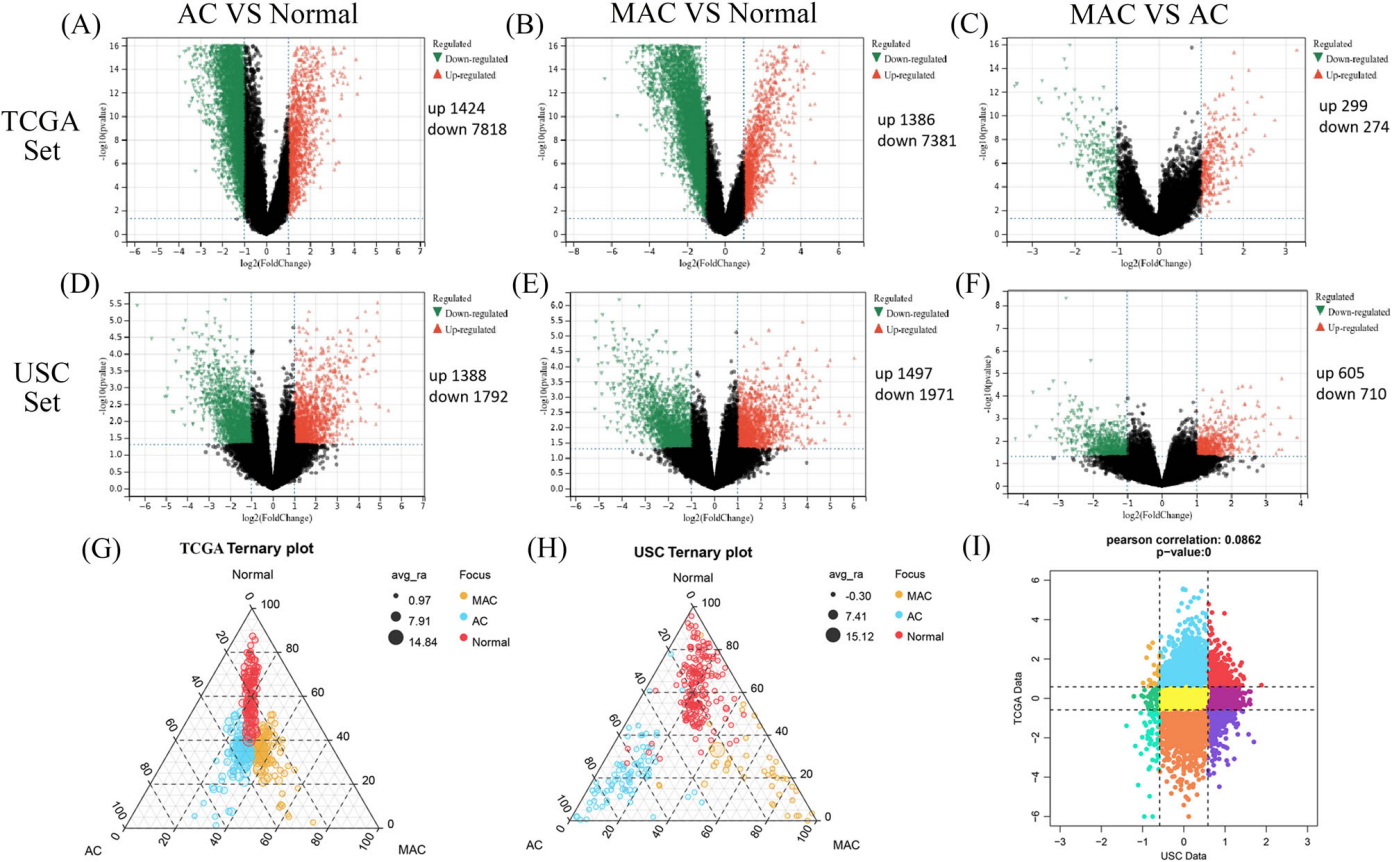
The analysis of differentially expressed genes (DEGs) among adenocarcinoma (AC), mucinous adenocarcinoma (MAC), and normal samples in TCGA and USC datasets. (A–C) The volcano plot of DEGs in AC versus Normal, MAC versus Normal, MAC versus AC samples in TCGA datasets. (D–F) The volcano plots of DEGs in AC versus Normal, MAC versus Normal, MAC versus AC samples in USC datasets. Red triangles indicate up‐regulated genes, and green triangles indicate down‐regulated genes as the legend. (G and H) The ternary plot of DEGs among AC, MAC, and Normal samples in TCGA and USC datasets. Genes more inclined to normal samples were marked in red, genes tend to AC samples were labeled in blue, and genes prone to MAC samples were tagged in yellow. (I) The 9‐quadrant diagram of the distribution of common DEGs between TCGA and USC datasets.

In 2011, Weinberg et al. summarized 10 cancer hallmarks, which become the cornerstone of tumour characteristic study.^8^ Thus, we analyzed the 10 hallmarks differences between AC and MAC. Results showed that evading growth suppressors and inducing angiogenesis appeared a marked weak activity. Nevertheless, reprogramming energy metabolism and tumour‐promoting inflammation were highly expressed. Unfortunately, we didn't observe a hallmark that was significantly different between MAC and AC (SM1, Figure S2A,C). In addition, we found some genes played an important role in multiple hallmarks, while others played the exclusive roles in single hallmark (SM1, Figure S2B,D).

According to Sadanandam et al. reported that CRCs could be classified into five cell phenotypes, which had distinct therapy response.^9^ We analyzed the cell subtype differences of MAC and AC both in TCGA and USC sets. Result showed that the samples of two datasets were well classified into five cell subtypes (Figure S3A,C), and the proportion of each subtype was different (Figure S3B,D). The Goblet‐like group accounted for the largest proportion in the MAC, followed by Stem‐like, and Ta was the least. On the contrary, the predominant cell subtype of AC was the Ta group, and the other four subtypes are relatively evenly distributed (Figure S3).

The consensus molecular subtype (CMS) is a new and important classification system for CRC.^10^ We also explored the association between CMS and MAC, and results showed that there was good consistency between subtype prediction and template features of TCGA and USC set (Figure 3A, D). The signal feature of each subtype was also studied. Results showed that the CMS1 group has obvious MSI characteristics, and the CMS2 group has distinct MSS, MYC, and cell cycle features, and differentiation signal was visible in CMS3, while TGF‐Beta and EMT signal was prominent in CMS4 (Figure 3B, E). Our results showed that AC group was enriched in CMS2 and CMS4 subtypes, and MAC group was enriched in CMS3 and CMS4 subtypes, especially in CMS3 (Figure 3C, F).

**FIGURE 3:**
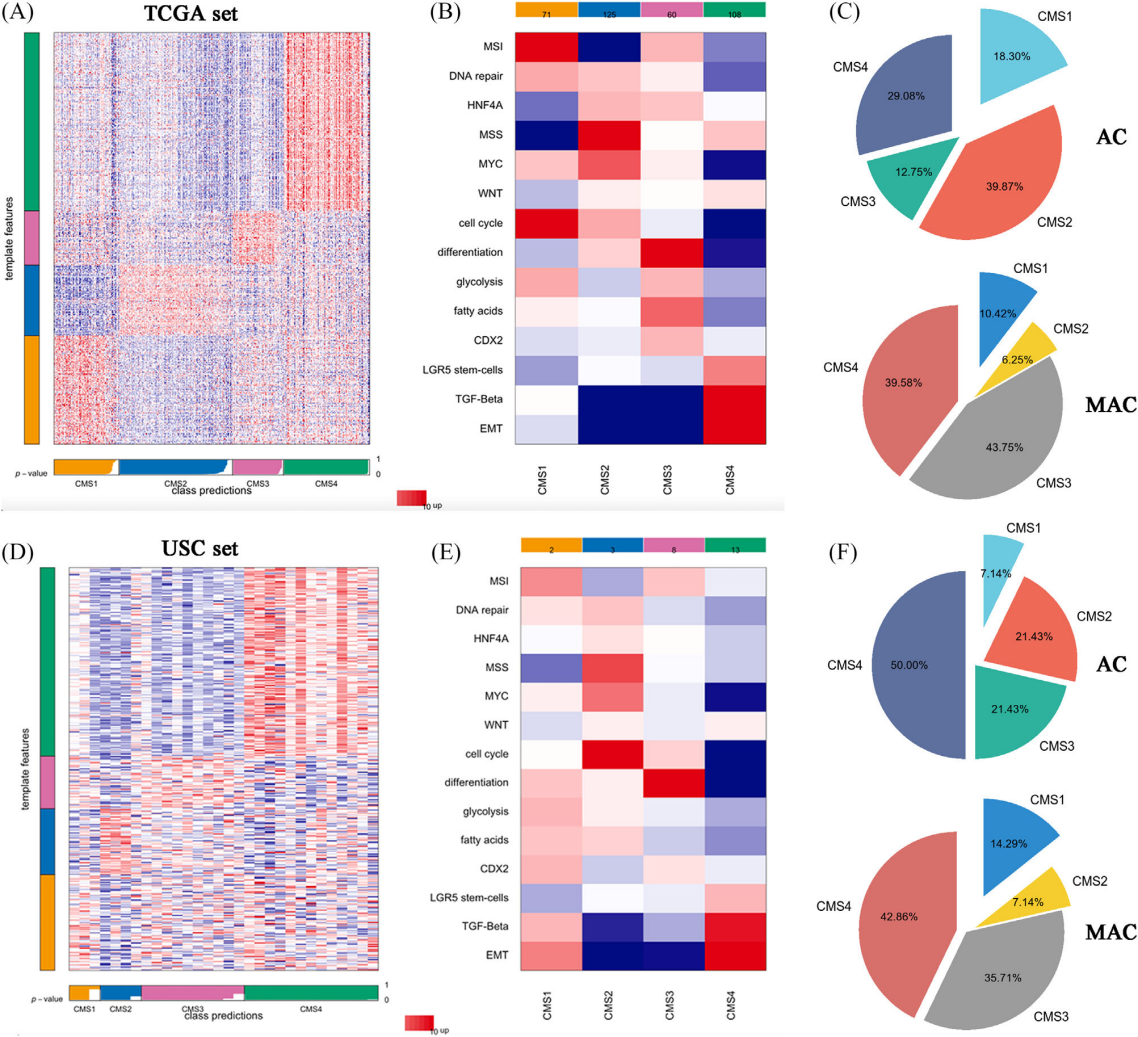
The consensus molecular subtype (CMS) subtype comparative analysis between mucinous adenocarcinoma (MAC) and adenocarcinoma (AC). (A and D) The CMS heatmap of top differentially expressed genes (DEGs) with shades of blue for down‐regulation and red for up‐regulation in TCGA and USC datasets. (B and E) The heatmap of pathways activity of each CMS subtype with shades of blue for low and red for high activity in TCGA and USC datasets. (C and F) The pie chart for each CMS subtype of AC and MAC samples in TCGA and USC datasets, respectively.

Previous studies indicated MAC was correlated with poor chemotherapy response and prognosis. To better understand the underlying molecular mechanisms, we analyzed GEO dataset of CRC drug resistance (GSE83129). GSEA was carried out to acquire the enriched pathway by our sequencing results and drug resistance dataset. Six shared GOBP pathways were enriched in MAC and chemotherapy non‐responder group (Figure 4A,B). The common genes interaction among these six GOBP pathways are shown in Figure 4C. We further found six intersection genes in GOBP①/GOBP③(Figure 4D), and 15 intersection genes in GOBP④/GOBP⑤ (Figure 4E). These 21 evolved drug‐resistant gene clusters might confer chemotherapy resistance to MAC.

**FIGURE 4:**
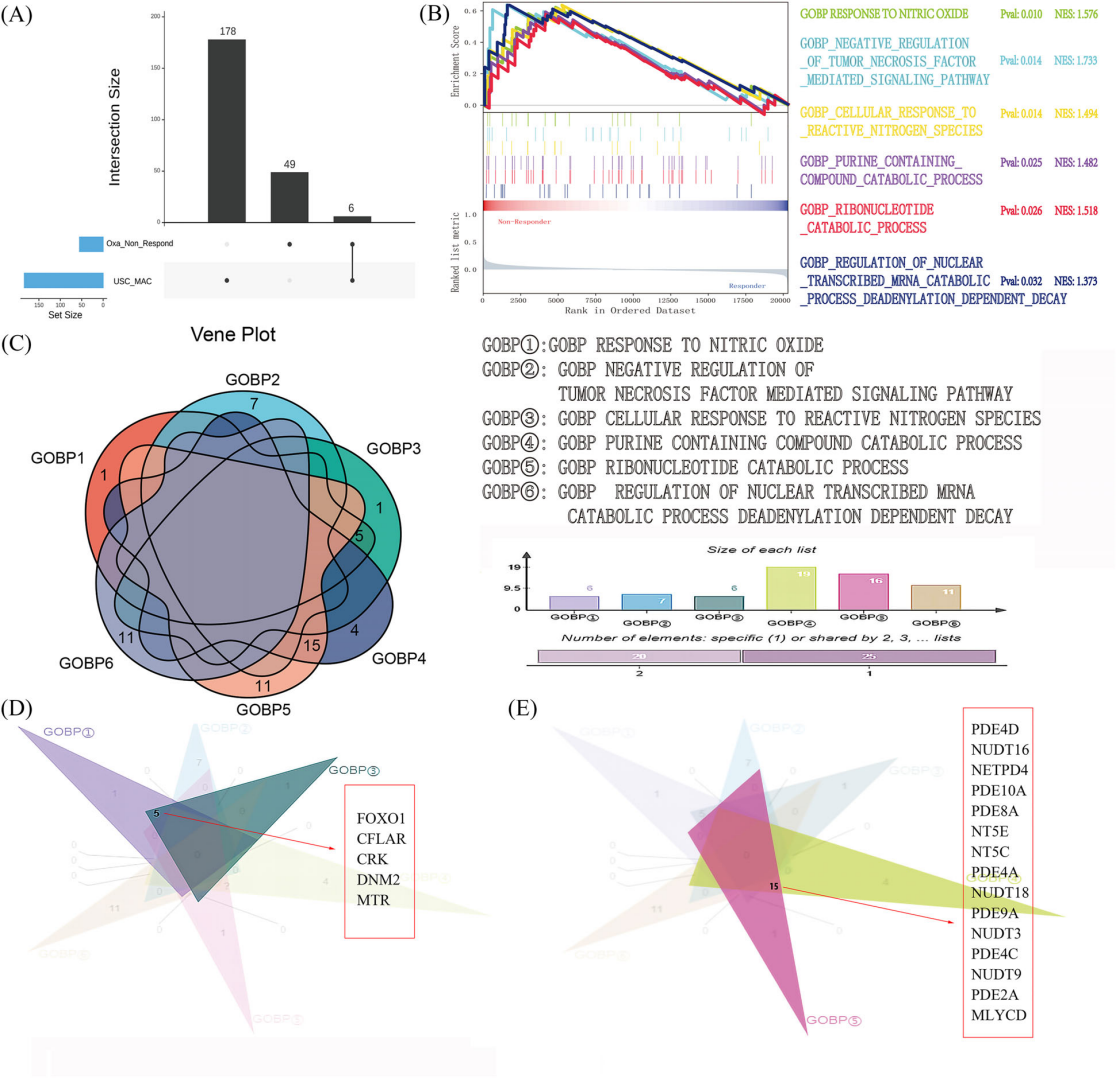
GSEA analysis of USC dataset and colorectal cancer (CRC) drug resistance dataset GSE83129. GSEA analysis showed the differentially enriched pathway in the mucinous adenocarcinoma (MAC)/adenocarcinoma (AC) samples and OXA_Responder/Non‐responder samples. (A) The upset plot showed the intersection pathways of MAC/AC samples and OXA_Responder/Non‐responder samples. (B) Shared enriched GO pathway by GSEA analysis. (C–E) Venn plots of the intersection of genes enriched in GO pathways. OXA, oxaliplatin.

In conclusion, this whole genome sequencing study preliminarily revealed the molecular and functional characteristics of MAC, as well as potential clinical value, which indicated that MAC tend to metabolic and mesenchymal phenotypes, contributing to worse prognosis and chemotherapy resistance.

## CONFLICT OF INTEREST STATEMENT

The authors declare no conflicts of interest.

## Supporting information

Supporting Information

Supporting Information

Supporting Information
