# Supplementary material for: Colorectal mucinous adenocarcinoma indicates a meaningful subtype: A whole genome sequencing study
Source: Clin Transl Med. 2023 Apr 26;13(4):e1246. doi: 10.1002/ctm2.1246 (PMC10131291; doi:10.1002/ctm2.1246)
Supplement: Supplementary file 1 — Supporting Information [file CTM2-13-e1246-s001.docx]

**Supplementary material**

**Materials and methods**

**Patients and samples**

There are total 40 cases of surgical specimens in this study, including 30 CRCs and 10 cases of non-tumor patients' colorectal tissues. Among the 30 CRC cases, there are 15 MACs and 15 ACs. Samples were retrospectively collected between January 2019 and December 2020 from the first affiliated hospital, University of South China (USC). All the CRC patients accepted colorectal radical resection and didn’t receive any preoperative radio-chemotherapy. The pathological diagnosis of surgical specimens met the criteria of the WHO, which was confirmed by two independent pathologists and the disparity was solved by discussing it with a senior pathologist. The detailed clinicopathological characteristics of MAC and AC patients were shown in Supplementary Table 1. This work was authorized by the ethics committee of the first affiliated hospital of USC (REC reference 202011181).

**Sample preparation and** **whole genome sequencing**

A total of 40 formalin-fixed and paraffin-embedded (FFPE) tissue samples were used in this study. The operating procedures of sample preparation for RNA extraction, and whole genome sequencing were followed the standard manufacturer’s protocol, which performed by our partner Gene^+^ company.

**Genomic mutation analysis**

The genomic mutation data of TCGA colorectal adenocarcinoma was downloaded from cBioPortal database (https://www.cbioportal.org/, (TCGA, PanCancer Atlas)). The top 15 most mutated genes of MAC and AC samples were selected and show as waterfall chart, and the mutation rate was calculated on the right side. The chi-Square test was used to compared the common 10 mutated gene between MAC and AC group, and p<0.05 considered to be statistically different.

**Differential Gene Expression Analysis**

The limma of R package was designed for differentially expressed genes (DEGs) analysis based on generalized linear models^1^. Here, we used the limma (version 3.40.6) to perform differential analysis to obtain DEGs between comparison groups and control groups as previously described^2^. Specifically, after the gene expression matrix we obtained, the multiple linear regression was performed by lmFit function, and further used the eBays function to construct difference comparison model. Finally, the p-value was corrected by the BH method. Genes with p value≤0.05 and |log2 fold change|≥1 are identified as DEGs.

**Function Enrichment Analysis**

The Gene Ontology (GO, http://geneontology.org/) is a concept that is designed as a formal representation of biological knowledge, which consists of three well knowledgeable portion: molecular function (MF), cellular component (CC), and biological process (BP)^3^. GO enrichment analysis could help us understand the biological functions of the DEGs. Kyoto Encyclopedia of Genes and Genomes (KEGG, http://www.kegg.jp/) is a database resource containing molecular interaction and reaction networks of human currently knowledge, which collected and exhibited by manually drawn pathway maps^4^. KEGG enrichment analysis could help us reveal the association pathways of DEGs. Hallmark gene sets are well-annotated biological states or processes, and they are coherently expressed signatures that excavated from MSigDB gene sets^5^. The Reactome (https://reactome.org) is a knowledge base that provides plentiful molecular details of DNA replication, signal transduction, transport, metabolism and other cellular processes^6^. Reactome functions as an archive of biological processes and a tool for discovering functional relationships of DEGs.

For function enrichment analysis of DEGs, we downloaded gene sets of items described above from the Molecular Signatures Database^7^ as a background, and then mapped DEGs into the background set using the R package clusterProfiler (version 4.3.0.991)^8^ to perform enrichment analysis to obtain gene set enrichment results. Function terms with p value <0.05 were considered to be significantly enriched.

**Cancer 10 Hallmark Enrichment Analysis**

In 2000, Weinberg *et al*. first summarized six hallmarks of cancer that provided an overall feature for conceptualizing the variety of tumors^9^. In 2011, they added another four hallmarks to depict the feature of cancers more fully that forming the 10 hallmarks of cancer as we know well^10^ for cancer 10 hallmark enrichment analysis, we first download the hallmark gene sets from CHG (Cancer Hallmark Genes, http://bio-bigdata.hrbmu.edu.cn/CHG/index.html) database^11^. Then the gene expression matrix data was inputted and the ssGSEA algorithm of GSVA(version 1.38.2)^12^ R package was used to calculate the enrichment score of every sample in each hallmark. Then the enrichment score matrixes were exhibited by heatmap through the ggplot2(version 3.3.6) R package. In addition, the DEGs between MAC and AC that enriched in each hallmark were shown by a circle plot.

**Cellular Phenotype and Consensus Molecular Subtypes (CMS) Analysis**

In 2013, Hanahan *et al*. classified colorectal cancer into five clinically relevant phenotypes, named : (i) goblet-like, (ii) enterocyte，(iii) stem-like，(iv) inflammatory， and (v) transit-amplifying (TA) respectively, each cell phenotype shares distinct characteristic^13^. The R package CRCassigner (version 1.0)^14^ was used for cellular phenotype analysis by setting PAM as 786 genes signature ("PAM786"). The results were visualized by heatmap, and the numbers of MAC and AC in each subtype were displayed by column diagram. In 2015, Tejpar *et al*. summarized the six independent classification systems coalescing into four CMS subtypes, named CMS1, CMS2, CMS3, and CMS4, with distinguishing features in each subtype^15^. The CMScaller (version 2.0.1)^16^ R package was used to analyze the CMS subtypes of the gene expression matrix. In addition, the activity of classic pathways such as MSI, DNA repair, HNF4A, etc. in each subtype also was explored by CMScaller. Finally, we counted the proportion of AC and MAC in each subtype and displayed them on a pie chart.

**Chemoresistance Analysis**

Colorectal chemoresistance datasets were searched in the GEO database. The dataset that contains sufficient resistant and non-resistant samples was considered for inclusion. Ultimately, GSE83129 was selected. Then, we employed the limma R package to analyze DEGs between resistance and non-resistance groups as described above. Finally, Gene Set Enrichment Analysis (GSEA) of DEGs was performed (http://www.broadinstitute.org/gsea/). The interacting enriched pathway between MAC vs AC and resistance vs non-resistance were considered as MAC chemoresistance pathway.

**Supplement reference**

[1]. Ritchie, M.E., et al., limma powers differential expression analyses for RNA-sequencing and microarray studies. Nucleic Acids Research, 2015. 43(7): p. e47.

[2]. Xu, Y.-H., et al., Identification of Candidate Genes Associated with Breast Cancer Prognosis. DNA and Cell Biology, 2020. 39(7): p. 1205-1227.

[3]. The Gene Ontology (GO) project in 2006. Nucleic Acids Research, 2006. 34(Database issue): p. D322-D326.

[4]. Kanehisa, M. and S. Goto, KEGG: kyoto encyclopedia of genes and genomes. Nucleic Acids Research, 2000. 28(1): p. 27-30.

[5]. Liberzon, A., et al., The Molecular Signatures Database (MSigDB) hallmark gene set collection. Cell Systems, 2015. 1(6): p. 417-425.

[6]. Jassal, B., et al., The reactome pathway knowledgebase. Nucleic Acids Research, 2020. 48(D1): p. D498-D503.

[7]. Liberzon, A., et al., Molecular signatures database (MSigDB) 3.0. Bioinformatics (Oxford, England), 2011. 27(12): p. 1739-1740.

[8]. Wu, T., et al., clusterProfiler 4.0: A universal enrichment tool for interpreting omics data. Innovation (Cambridge (Mass.)), 2021. 2(3): p. 100141.

[9]. Hanahan, D. and R.A. Weinberg, The hallmarks of cancer. Cell, 2000. 100(1): p. 57-70.

[10]. Hanahan, D. and R.A. Weinberg, Hallmarks of cancer: the next generation. Cell, 2011. 144(5): p. 646-674.

[11]. Zhang, D., et al., CHG: A Systematically Integrated Database of Cancer Hallmark Genes. Frontiers In Genetics, 2020. 11: p. 29.

[12]. Hänzelmann, S., R. Castelo, and J. Guinney, GSVA: gene set variation analysis for microarray and RNA-seq data. BMC Bioinformatics, 2013. 14: p. 7.

[13]. Sadanandam, A., et al., A colorectal cancer classification system that associates cellular phenotype and responses to therapy. Nature Medicine, 2013. 19(5): p. 619-625.

[14]. Ragulan, C., et al., Analytical Validation of Multiplex Biomarker Assay to Stratify Colorectal Cancer into Molecular Subtypes. Scientific Reports, 2019. 9(1): p. 7665.

[15]. Guinney, J., et al., The consensus molecular subtypes of colorectal cancer. Nature Medicine, 2015. 21(11): p. 1350-1356.

[16]. Eide, P.W., et al., CMScaller: an R package for consensus molecular subtyping of colorectal cancer pre-clinical models. Scientific Reports, 2017. 7(1): p. 16618.
