# Supplementary material for: Colorectal mucinous adenocarcinoma indicates a meaningful subtype: A whole genome sequencing study
Source: Clin Transl Med. 2023 Apr 26;13(4):e1246. doi: 10.1002/ctm2.1246 (PMC10131291; doi:10.1002/ctm2.1246)
Supplement: Supplementary file 2 — Supporting Information [file CTM2-13-e1246-s002.docx]

**Supplementary material 1(SM1)**

**Additional description**

Colorectal cancer (CRC) is divided into numerous histological subtypes, the non-specific adenocarcinoma (AC) is the most common subtype and occurs in approximately 85% of entire CRC patients. The second most common histological subtype is mucinous adenocarcinoma (MAC), which accounts for 10-15% of all CRCs. Research reported that MAC has distinct characteristics to AC, such as female propensity, more proximal colon location, more-advanced tumor stage, more common history of inflammatory bowel diseases (IBD) and radiotherapy, as well as different molecular features. The major molecular characteristics of MAC include a high frequency of microsatellite instability (MSI) and CpG island methylator phenotype-high (CIMP-H), high KRAS, PIK3CA mutation and MUC2 overexpression. However, the underlying molecular mechanism for their difference is still not clear. Based on this, we focused MAC research. Our previous studies found that the overall survival (OS) of the MAC group was poorer than that of the AC group, but cancer-specific survival (CSS) was similar between the two groups^1^. In addition, we found surgery could improve the prognosis of colon mucinous adenocarcinoma with liver metastases^2^, and hemicolectomy does not provide survival benefit for right-sided mucinous colon ddenocarcinoma^3^. What’s more, we found endoscopy biopsy is not efficiency enough for diagnosis of mucinous colorectal adenocarcinoma^4^.

In order to more deeply study on MAC, we conducted RNA-seq research on CRC samples in this article. Through combined with TCGA-COAD transcriptomics and clinical data, we observed that MAC was distinct with AC in genomic, transcriptome, CMS subtypes, chemotherapy response etc.

To uncover the potential distinct gene expression profile of MAC compared to AC which might result in functional differences, we performed a functional enrichment analysis of DEGs from MAC vs AC groups that were discovered by USC set and TCGA set. Through GO analysis, we found the biological processes (BP) of these DEGs were significantly enriched in the positive regulation of the prostaglandin biosynthetic process, positive regulation of the unsaturated fatty acid biosynthetic process, and regulation of ERK1 and ERK2 cascade, etc. The cellular component (CC) analysis results showed that these DEGs assembled in the extracellular region part, extracellular region, and extracellular exosome, etc, which were consistent with a mucoid phenotype of MAC. The molecular function (MF) analysis indicated that these DEGs mainly correlated with inorganic anion exchanger activity, solute: sodium symptom activity, etc, affecting transportation a wide range of drugs (Figure S1A).

The KEGG pathway analysis elucidated that these DEGs were enriched in metabolism related pathways, such as anachronism acid metabolism, phenylalanine, tyrosine and tryptophan biosynthesis, etc, organismal systems related pathways, such as bile secretion, complement and coagulation cascades, and environmental information processing pathways, such as calcium signaling pathway and neuroleptic ligand-receptor interaction (Figure S1B).

For hallmarks enrichment analysis, the results showed that these DEGs enriched in estrogen response late, coagulation, pancreas beta cells, apical surface, etc. (Figure S1C). Finally, the reactome analysis results indicated DEGs assembled in the regulation of signaling by nodal, defective galnt3 causes familial hyperphosphatemic tumoral calcinosis hftc, and defective cigaltic causes tn polyagglutination syndrome tnps, etc (Figure S1D).

Through GSEA analysis of 10 cancer hallmarks, we found evading growth suppressors, evading immune destruction, genome instability and mutation, and inducing angiogenesis appeared a weak activity. While, reprogramming energy metabolism, enabling replicating immortality, sustaining proliferative signaling, tumor-promoting inflammation, activating invasion and metastasis, and resisting cell death well highly expressed both in MAC and AC groups of the USC and TCGA sets (Figure S2A, C). In addition, we analyzed the distribution of DEGs between MAC and AC in 10 hallmarks. We can see that some DEGs play an important role in multiple hallmarks, such as PRKCG, PTCS2, FGFR2, IL1B, etc. Nevertheless, PKLR, SLC11A1, UGT2B4 and other genes played exclusive roles in reprogramming energy metabolism, resisting cell death, genome instability and mutation, and other hallmarks respectively (Figure S2B, D). These results indicated that although the 10 hallmarks are not obviously different between MAC and AC, the internal of 10 hallmarks is distinct. CRC appears to be a “hot” tumor in proliferation, whereas a “cold” tumor in immunization.

**Supplement reference**

1. Zhang Y, Chen Y, Huang J, et al. Mucinous histology is associated with poor prognosis in locally advanced colorectal adenocarcinoma treated with postoperative first-line adjuvant chemotherapy: A systematic review and meta-analysis. *Eur J Surg Oncol*. 2022;48(10):2075-2081. doi:10.1016/j.ejso.2022.06.024

2. Huang J, Chen G, Liu H, et al. Surgery improves the prognosis of colon mucinous adenocarcinoma with liver metastases: a SEER-based study. *BMC Cancer*. 2020;20(1):908. doi:10.1186/s12885-020-07400-4

3. Huang J, Huang Q, Tang R, et al. Hemicolectomy Does Not Provide Survival Benefit for Right-Sided Mucinous Colon Adenocarcinoma. *Frontiers In Oncology*. 2020;10:608836. doi:10.3389/fonc.2020.608836

4. Xiao S, Huang J, Zhang Y, et al. Endoscopy biopsy is not efficiency enough for diagnosis of mucinous colorectal adenocarcinoma. *Discov Oncol*. 2021;12(1):44. doi:10.1007/s12672-021-00443-4
