# Supplementary material for: Colorectal mucinous adenocarcinoma indicates a meaningful subtype: A whole genome sequencing study
Source: Clin Transl Med. 2023 Apr 26;13(4):e1246. doi: 10.1002/ctm2.1246 (PMC10131291; doi:10.1002/ctm2.1246)
Supplement: Supplementary file 3 — Supporting Information [file CTM2-13-e1246-s003.doc]

**Supplementary material**

**Supplementary Tables**

Supplementary Table 1. The baseline characteristics of MAC and AC patients.

|  | **Sex** | **Age** | **Location** | **Size** | **pT stage** | **pN stage** | **MMR** | **MVI/PNI** |
| --- | --- | --- | --- | --- | --- | --- | --- | --- |
|  |  |  |  |  |  |  |  |  |
| **MAC 1** | Female | 76 | Proximal | 7 | 3 | 0 | dMMR | Absent |
| **MAC 2** | Male | 53 | Distal | 10 | 2 | 0 | pMMR | Absent |
| **MAC 3** | Female | 60 | Proximal | 7 | 3 | 1 | NA | Absent |
| **MAC 4** | Female | 72 | Proximal | 2.5 | 4 | 0 | pMMR | Absent |
| **MAC 5** | Female | 69 | Proximal | 8.6 | 4 | 0 | pMMR | Absent |
| **MAC 6** | Female | 70 | Distal | 6 | 3 | 1 | pMMR | Absent |
| **MAC 7** | Male | 66 | Proximal | 8 | 3 | 0 | pMMR | Present |
| **MAC 8** | Female | 51 | Proximal | 5 | 3 | 0 | pMMR | Absent |
| **MAC 9** | Female | 75 | Proximal | 8 | 3 | 0 | NA | Absent |
| **MAC 10** | Male | 33 | Rectal | 5 | 4 | 2 | pMMR | Present |
| **MAC 11** | Female | 44 | Proximal | 10 | 3 | 0 | dMMR | Absent |
| **MAC 12** | Male | 57 | Proximal | 8.5 | 3 | 0 | pMMR | Absent |
| **MAC 13** | Male | 56 | Proximal | 10 | 4 | 0 | dMMR | Absent |
| **MAC 14** | Male | 50 | Rectal | 4 | 4 | 0 | pMMR | Absent |
| **MAC 15** | Male | 83 | Proximal | 6.5 | 3 | 0 | pMMR | Absent |
| **AC 1** | Male | 65 | Rectal | 3.5 | 3 | 0 | pMMR | Absent |
| **AC 2** | Female | 72 | Distal | 3.5 | 3 | 1 | pMMR | Absent |
| **AC 3** | Female | 64 | Rectal | 7.5 | 3 | 0 | pMMR | Absent |
| **AC 4** | Female | 52 | Distal | 4.5 | 3 | 0 | pMMR | Absent |
| **AC 5** | Female | 68 | Proximal | 6.5 | 3 | 0 | pMMR | Absent |
| **AC 6** | Female | 61 | Proximal | 7 | 2 | 0 | pMMR | Absent |
| **AC 7** | Female | 64 | Proximal | 6 | 4 | 0 | pMMR | Absent |
| **AC 8** | Female | 62 | Distal | 2.5 | 2 | 0 | pMMR | Absent |
| **AC 9** | Female | 66 | Rectal | 6.5 | 4 | 0 | pMMR | Absent |
| **AC 10** | Male | 53 | Proximal | 5.5 | 2 | 0 | pMMR | Absent |
| **AC 11** | Female | 49 | Distal | 7.5 | 3 | 0 | pMMR | Absent |
| **AC 12** | Female | 75 | Distal | 4.5 | 4 | 0 | NA | Absent |
| **AC 13** | Female | 48 | Distal | 4 | 3 | 0 | NA | Absent |
| **AC 14** | Female | 67 | Distal | 4 | 2 | 0 | dMMR | Absent |
| **AC 15** | Male | 64 | Distal | 3.5 | 4 | 1 | pMMR | Present |

Supplementary Table 2. The clinical characteristics of MAC and AC of TCGA-COAD samples.

|  | AC (398 cases) | MAC (69 cases) | P |
| --- | --- | --- | --- |
| **Sex** |  |  |  |
| Female | 188 | 34 |  |
| Male | 210 | 35 | 0.754 |
| **Age (years)** | 67±13 | 67±15 | 0.873 |
| **Pathologic_T** |  |  |  |
| T1+T2 | 81 | 10 |  |
| T3+T4 | 316 | 59 |  |
| Tis | 1 | 0 | 0.476 |
| **Pathologic_N** |  |  |  |
| N0+ N1 | 328 | 54 |  |
| N2 | 70 | 15 | 0.409 |
| **Pathologic_M** |  |  |  |
| M0 | 294 | 48 |  |
| M1 | 56 | 8 |  |
| Mx+NA | 48 | 13 | 0.289 |
| **Tumor_stage** |  |  |  |
| StageⅠ+Ⅱ | 222 | 38 |  |
| StageⅢ+Ⅳ | 165 | 31 |  |
| NA | 11 | 0 | 0.353 |
| **MSI** |  |  |  |
| Yes | 11 | 3 |  |
| No | 68 | 15 |  |
| NA | 319 | 51 | 0.473 |
| **dMMR** |  |  |  |
| Yes | 54 | 6 |  |
| No | 250 | 34 |  |
| NA | 94 | 29 | 0.05 |
| **Chemotherapy effect** | 135 cases | 22 cases |  |
| Response | 62 | 10 |  |
| Resistance | 23 | 6 |  |
| NA | 50 | 6 | 0.454 |

**Supplementary Figures**


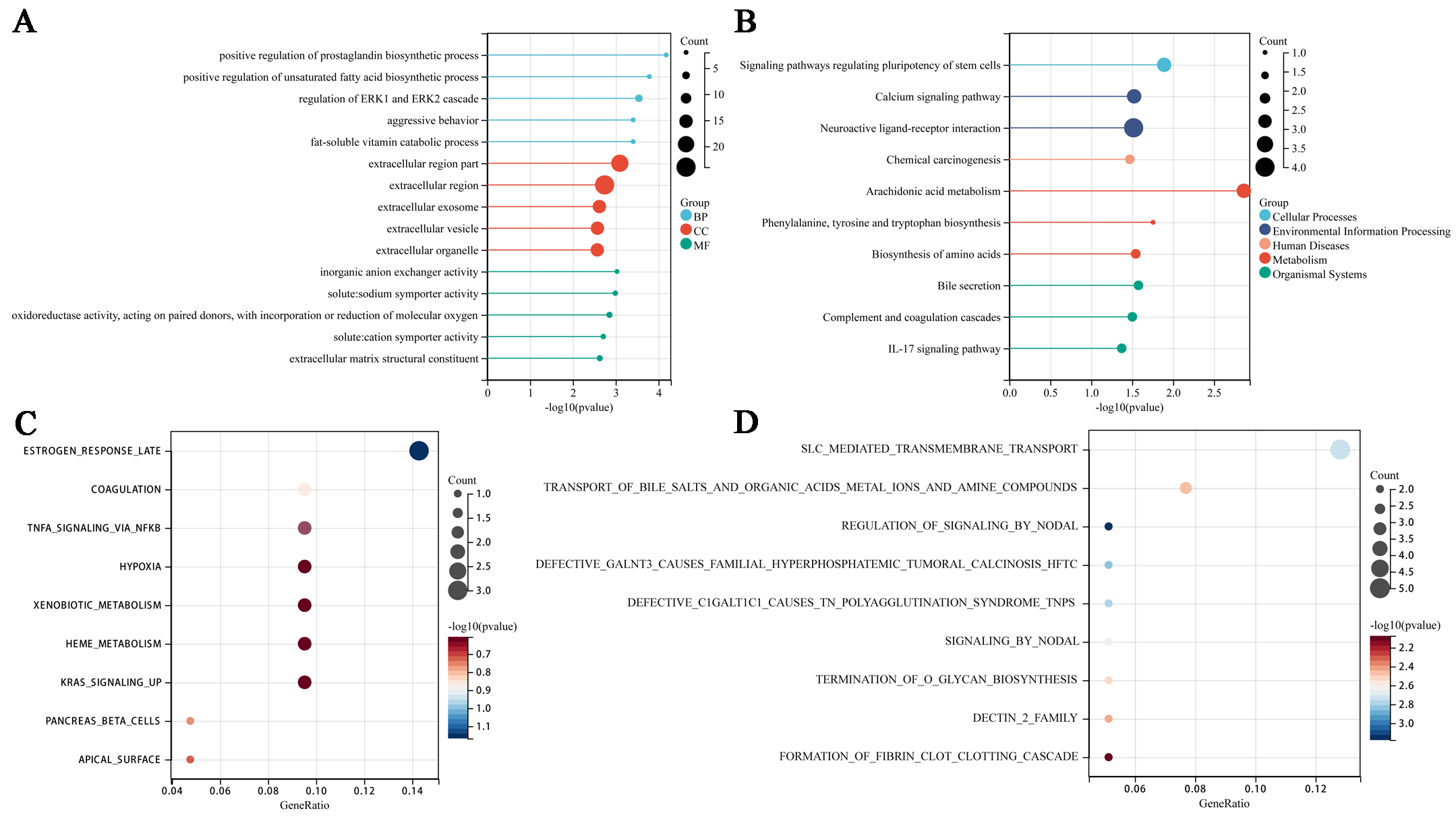


Supplementary Figure 1. The function enrichment analysis of common DEGs of MAC vs AC in TCGA and USC datasets. (A) The GO enrichment analysis of common DEGs, comprised BP (blue), CC (red), and MF (green). (B) The KEGG enrichment analysis of common DEGs. (C) The hallmarks enrichment analysis of common DEGs. (D) The Reactome enrichment analysis of common DEGs.


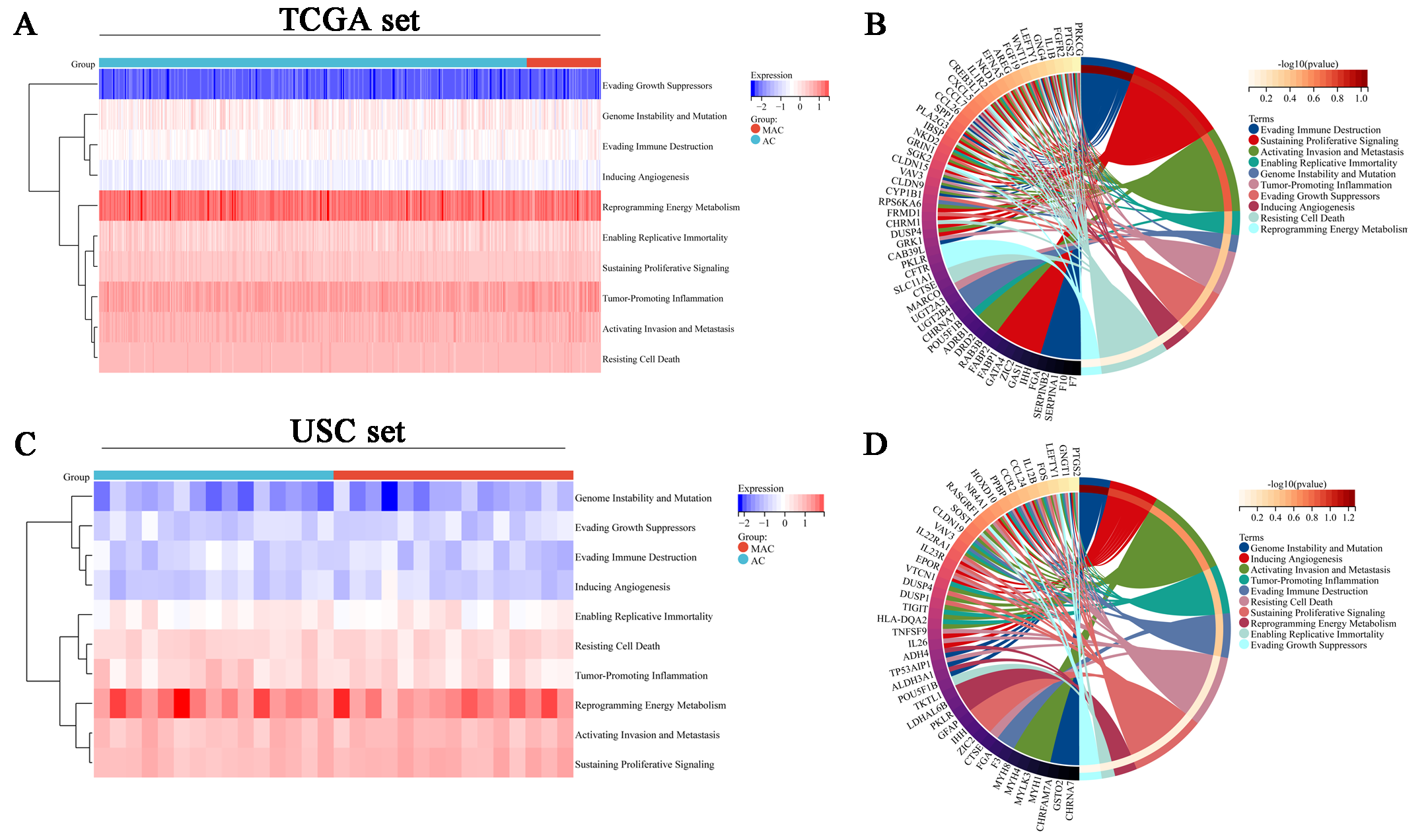


Supplementary Figure 2. The 10 cancer hallmarks characteristic analysis of MAC and AC. (A, C) The heatmap of 10 cancer hallmarks in TCGA, and USC dataset respectively. Each column denotes an individual tumor and each row represents the individual hallmarks. AC samples are marked in blue, and MAC samples are marked in red. The depth of color indicates the level of expression. (B, D) The circle map of the DEGs distribution in each hallmark in TCGA, and USC datasets respectively. Different colors represent different hallmarks and the depth of color indicates the p value as shown in the legend.


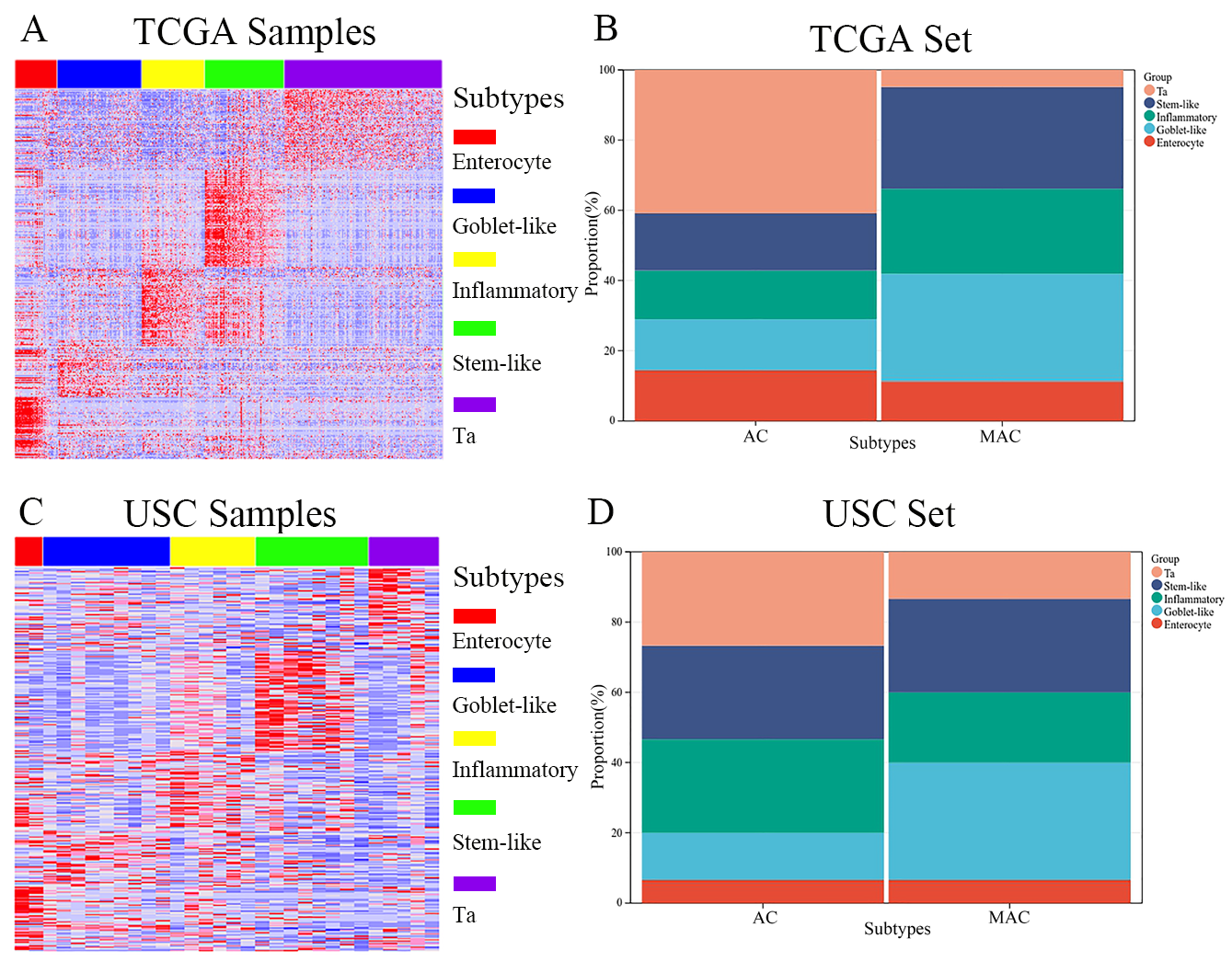


Supplementary Figure 3. The cell phenotype comparative analysis between MAC and AC. (A, C) The CRC cell phenotype heatmap of CRC samples of TCGA, USC dataset respectively. Each column denotes an individual tumor. (B, D) The proportion histogram of each cell phenotype of AC and MAC samples in TCGA, and USC datasets respectively.
